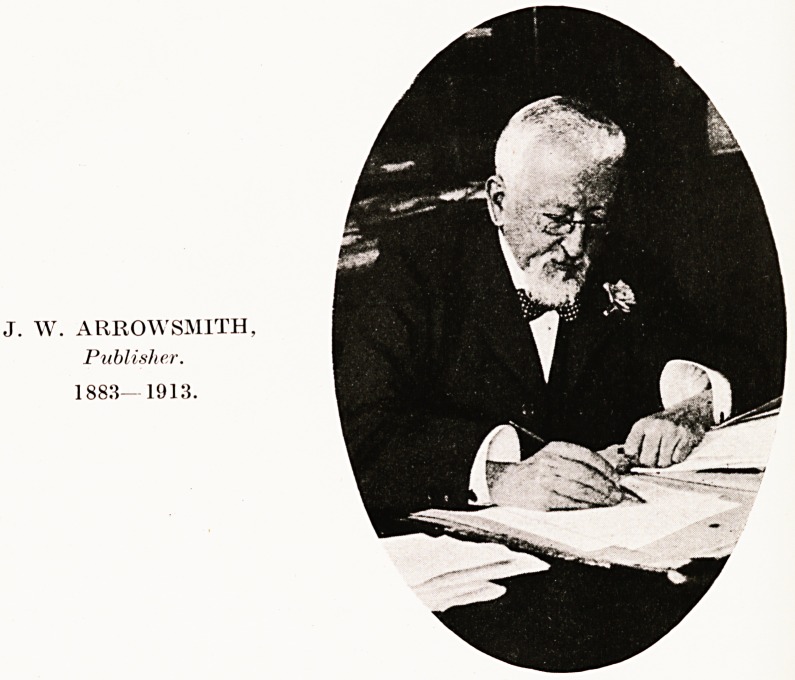# The History of the Bristol Medico-Chirurgical Journal

**Published:** 1933

**Authors:** Patrick Watson-Williams

**Affiliations:** Assistant Editor 1900–1912, Editor 1912–1926


### THE HISTORY OF "THE BRISTOL MEDICO-CHIRURGICAL JOURNAL."

BY

PATRICK WATSON-WILLIAMS, M.D.,

*Assistant Editor 1900–1912, Editor 1912–1926.*

---

THE publication of Volume I in 1883 dates our *Journal's* birth, and this, its fiftieth year of an unbroken career, is an occasion for congratulations. We can look not only forward to its future with confidence, but back to its past with legitimate pride in its parentage and early years. The Society had already published in 1878 a volume of *Transactions*, embodying the Proceedings of the Society for 1874–78, under the auspices of the energetic young secretary, Dr. R. Shingleton Smith. One is strongly tempted to dwell on this volume, which contained many beautiful drawings and original scientific contributions.

The actual inception of the *Journal* has been recorded by Mr. L. M. Griffiths, for seventeen years Assistant Editor.[1] After referring to the preceding *Transactions*, he continued: "Another four years passed (*i.e.* 1882) and some members of the Committee thought the time had arrived for the publication of a second volume of *Transactions*, covering that period. Feeling that such a retrospect was at its best by no means satisfactory, and knowing well the ability of my professional brethren in the district, I had the temerity to propose in Committee that a publication

[1] Presentation to Mr. L. M. Griffiths, June, 1900, vol. xviii., p. 97.

with a more frequent periodicity, and having the nature of a Journal, should be attempted. This proposition received the support only of Greig Smith and myself. But when the question introduced by Dr. Beddoe was brought before a general meeting of the Society, it was determined *nemine contradicente* that the effort should be made."

The first number of the *Bristol Medico-Chirurgical Journal* appeared in July, and the second, completing the first volume, in December, 1883. The Editor was J. Greig Smith; Assistant Editor, L. M. Griffiths; published by Messrs. J. W. Arrowsmith; 270 pages, numerous illustrations from drawings on stone and photographic prints pasted in. The pale-green cover that most of our readers can still remember bore the lines of Lucilius:—

> "Scire est nescire, nisi id me
> Scire alius sciret."

The contents of the *Journal* are being presented in the forthcoming fifty-year Index; here it is possible only to recall certain outstanding contributions. But the first volume deserves an ampler note, showing that our foundations were well and truly laid.

It opens with an article by R. Shingleton Smith: "The Proofs of the Existence of a Phthisical Contagion," introduced as "a subject of peculiar interest to the medical profession of this district, inasmuch as it was here that our late colleague, Dr. William Budd, startled the profession by announcing his belief that phthisis would be found to be contagious."[1] E. Markham Skerritt follows, with clinical records and investigations pointing to the dominance of the factors of heredity and suitable soil as evidence *against* the contagiousness of phthisis (22 pages).

[1] See page 172 of this issue.

Then came G. Munro Smith, on "The Cardiograph" (*i.e.* Sphygmomanometer); W. H. Harsant, "Surgical Notes"; Case Reports by E. Long Fox, G. Thompson, J. Fenton Evans, J. Fuller, J. E. Shaw, and five by J. Greig Smith—the latter and many others beautifully illustrated. Book Reviews and "Medical Extracts" complete the first number.

The second number opens with "Placenta Prævia," by J. G. Swayne; then "An Epidemic of Tetanus," by A. Sheen (Cardiff); "Medical Posture," by J. Kent Spender (Bath); "Perforating Ulcer of the Stomach," by Nelson C. Dobson; "Retinoscopy," by A. Prowse; and "Clinical Records." Next, notes on the University College Medical School; Report of the Faculty (90 students, 38 new entrants); and Dr. Beddoe's Address (10 pages) at the distribution of prizes. Surely it is one of the uses of a Journal to note the progress of our Medical School and the apprentice footsteps of its sons.

After the first volume the *Journal* appeared quarterly. In 1884 we are introduced to cocaine, the new local anæsthetic. Notes by Dr. Fenton Evans on a young woman who had thrown herself over the Clifton Suspension Bridge are particularly interesting as the only case in which an attempt at suicide from this bridge has failed. The writer was in residence when the patient was brought into the Royal Infirmary, collapsed and pulseless, and hence was particularly interested to learn quite recently that she was alive and well. In Volume IV (1886) there began to appear "Scraps," collected by the Assistant Editor.

In 1891 an Editorial Committee was appointed. The members were: Dr. Barclay Baron, Dr. Michell Clarke, Mr. F. R. Cross, Mr. W. H. Harsant. The Committee met for the first time on January 28th at 28 Berkeley Square.

1892 is memorable in the history of the *Journal*, because Dr. R. Shingleton Smith was appointed Editor in succession to Mr. J. Greig Smith, whose resignation the Society accepted with great regret. In the December number an account is given of the opening, by Sir Andrew Clark, on 16th November, of the handsome new wing of University College, assigned to the Bristol Medical School. The cost of the Library Building and Council Chamber was mainly defrayed by the subscriptions of our members. We refer later (p. 161) to the foundation of the Library. Of supreme interest is Mr. Augustin Prichard's account of the "Early History of the Bristol Medical School." In this year half-tone electros were first used for illustrations; they have the merit of simplicity and cheapness, but one cannot help regretting the beautiful hand-drawn illustrations of the earlier volumes.

Volume XII, 1894, is particularly memorable for its record of the Annual Meeting here of the British Medical Association, under the Presidency of Dr. Edward Long Fox. The *Journal* recalls the first anniversary meeting here of the "Provincial Medical and Surgical Association" in 1833,[1] and the meeting of the British Medical Association in 1863.

The Editorial Committee held their meetings in the University College from 25th July, 1893, onwards. Mr. L. M. Griffiths's energies had so added to his self-imposed work that in 1895 Dr. Bertram Rogers was appointed Editorial Secretary. In 1898 he found himself unable to spare the time required for the work of Secretary, though he consented to remain on the Committee, and Mr. James Taylor was appointed Editorial Secretary.

At the one hundred and twelfth meeting of the

[1] See p. 181 of this issue.

Committee on 26th December, 1899, Mr. L. M. Griffiths announced his resignation, to the deepest regret of the Committee. The members recorded their high appreciation of his indefatigable and self-denying services, and particularly requested him to continue his " Scraps " ; and the Society presented him with an Address of Appreciation and other substantial marks of their sense of gratitude. Yet none but those who, like the writer in after years, were engaged on the production of the *Journal* ever really learnt how much of our success was due to Griffiths. In all arrangements for the production, for advertisements, for reviews, for " exchanges," and in the arduous secretarial duties he was untiring.

On 30th January, 1900, Dr. P. Watson-Williams was elected Assistant Editor. He proposed and had permission to arrange for Editorial Notes on questions of special interest. The movement then afoot with a view to the establishment of a University of Bristol was the subject of some of the earliest of these Notes.

The Presidential Address for this year by Dr. Lionel Weatherley, " A Plea for Sanatoria for Poor Consumptives," is particularly memorable in connection with the subsequent establishment of the Winsley Sanatorium. It was largely due to Dr. Weatherley's moving appeals that this particular object of the Three Counties Branch of the National Association for the Prevention of Consumption was attained. But the movement had already been initiated by some members of the Committee of the Medico-Chirurgical Society under the leadership of Dr. E. Long Fox, when he called a public meeting at Clifton on 6th July, 1899. At this meeting the Three Counties Branch for Gloucester, Wilts and Somerset was inaugurated. It may be noted that the donations of private charity towards the land and buildings of Winsley Sanatorium between 1899 and 1912 exceeded £21,000, all of which has passed into the control of the Bristol Corporation—a sign of the times. The *Journal* records at some length the activities of the Three Counties Branch.

Volume XIX, for 1901, is adorned with a frontispiece of her late Majesty Queen Victoria at the opening of the Jubilee Convalescent Home (13th November, 1899). The *Journal* minutes record that this was provided by the Editor at his own cost ; and also that " financial difficulties have restricted the efforts of the Editor and his Committee." Financial straits were constantly matters for consideration for many years.

The Long Fox Lecture was instituted in memory of Edward Long Fox, who died in the year 1902, " to encourage research in subjects concerned with the science of medicine." These lectures, which are representative of original work and clinical investigation, have mostly been published exclusively in our *Journal*, and hence have especial claims to notice in our history. Dr. Edward Long Fox was physician to the Bristol Royal Infirmary, 1857–77, and Lecturer in Medicine and Pathological Anatomy at the Bristol Medical School.

The first lecture was delivered on 4th November, 1904, by Dr. John Beddoe, F.R.S., on " The Ideal Physician." He referred to Dr. Long Fox's worth and work which was " rewarded with love, honour and troops of friends," and recalled one of his less-known services to our city in the typhus epidemic which broke out shortly after Dr. Fox had begun practice. " We had no Medical Officer of Health . . . but Long Fox created an organization to meet the situation and devoted himself personally to the work." As more

L. M. GRIFFITHS,
*Assistant Editor.*
1883—1899.

J. W. ARROWSMITH,
*Publisher.*
1883—1913.

directly concerning our *Journal* we may recall that Dr. Long Fox made a successful and organized effort amongst his colleagues and friends to raise the amount required for the new Medical School building which, when erected, housed the Medical Library as well as what is now the Senate Room, where all the meetings of the Society were held for years.

In 1907 the Monotype machine was first used to set the type for the *Journal.*

In 1912 Dr. R. Shingleton Smith resigned the Editorship after twenty years' service. Dr. P. Watson-Williams, Assistant Editor since 1900, was elected to fill the vacancy, and Dr. J. A. Nixon became Assistant Editor. The other members of the Committee, Dr. J. Michell Clarke, Dr. James Swain and Mr. J. Lacy Firth, continued their valued services, to the great assistance of the new Editor. Dr. Fortescue-Brickdale was appointed Editorial Secretary.

The *Journal* lost one of its best friends early in 1913 by the death of Mr. J. W. Arrowsmith, the founder of our firm of publishers, whose personal devotion to the production of the *Journal* from its earliest days onwards was extremely helpful.

The difficulties which arose during the War cannot be passed in silence. At the outset the Editorial Committee carried on as usual, but as time wore on their work was carried on with ever-increasing difficulty. Captain Fortescue-Brickdale was ordered abroad in 1915, Dr. Nixon went overseas with the Sixty-first Division in 1916, and Colonel James Swain was on duty as Advisory Consultant Surgeon to the Army. Colonel Michell Clarke and Captain Lacy Firth, who alone remained of the Editorial Committee, were very much occupied with their arduous duties with the Second Southern Hospital.

The writer, at that time Editor, apologizes for the obvious shortcomings of the *Journal* during the later years of the War and for some time subsequently. For these shortcomings he seeks some excuse in that his work as Consulting Aural Surgeon to the Southern Command occupied an overwhelming proportion of his time. Among other unforeseen difficulties was the increase in the cost of paper, which was only overcome by the happy chance afforded of making a large purchase in advance. The Editor took this responsibility himself; otherwise even the rather slender war numbers would have been impossible. As it was, from 1915 to 1922 only twenty numbers could be published, two or three for each year. Volume XXXVI comprises one number for 1918 and two for 1919. Among other interesting features of the "War" *Journal* are a complete nominal roll of the staff of the Second Southern General Hospital and a record of Honours conferred on local colleagues.

By the death of Lieut.-Col. J. Michell Clarke in the year 1918, whilst still on war service, the Editorial Committee of the *Journal* lost its senior member, who had rendered us devoted service since 1891. It was a severe blow, for his guidance was always to be trusted.

In Vol. XXXVII, 1920, is an Editorial Note on the proposed amalgamation of Bristol Hospitals.

In 1924 the Editorial Committee sustained a further loss by the resignation of Colonel James Swain, C.B., C.M.G., whose collaboration had continued ever since 1897, and throughout this lengthy period had been of the greatest value, particularly in regard to surgical subjects.

Volume XLII, No. 156 (1925) was made a special number, profusely illustrated, to mark the Royal opening of the new University Buildings. It opens with a short survey of the evolution of the early conception of a University in Bristol, first suggested nearly three centuries ago by Dr. Dell, Cromwell's chaplain, and of the subsequent long-delayed steps which culminated in the actual foundation of the University in 1909. The various views of the University Buildings and of the Royal Infirmary and General Hospital, together with many interesting details relating to the work of the several departments, will be valued as a record of historical value. The entire number contained many illustrations and was printed on art paper and bound in a special cover. We believe that it worthily commemorates a great occasion.

The year 1926 opened with the *Journal* in larger page and type, a long-cherished ambition till then, perforce, delayed. The cover was changed to the dark neutral tint of the binding of the special number, which has remained in use up to the present, and the motto disappeared from it. With the publication of this issue the Editor resigned his charge, having completed twenty-six years of service, first as Assistant Editor since January, 1900, and as Editor since December, 1912.

Dr. J. A. Nixon was elected Editor in March, 1926, and Dr. Carey F. Coombs Assistant Editor. Mr. Lacy Firth resigned from the Editorial Committee in April, after many years of valued work. The vacancies in the Committee were filled by the election of Mr. E. Watson-Williams and of Mr. A. E. Iles, who had just been chosen for the newly-created and responsible office of Treasurer to the Society. The innovation of bringing the finances of the *Journal* into the same hands as those of the Society has proved of the greatest value. That Dr. A. L. Flemming consented to remain as Editorial Secretary was most helpful to the Editor and his colleagues, and as Dr. Flemming was this year President of the Society, it was no light service. His Presidential Address on "Anæsthesia" comprised a most interesting and scholarly review of the earlier developments in anæsthesia, of course including a reference to Humphry Davy's discovery of the properties of nitrous oxide gas while he was in residence in Bristol. But Dr. Flemming forgot to tell us anything of his own initiation of the open-ether technique in 1896; most of the older practitioners recall the semi-asphyxiation that the ether bag involved. It was Hewitt, the most notable anæsthetist of his day, who referred to the open-ether light anæsthesia we know so well as the "Bristol method." For many years it remained essentially the Bristol method, though it has now been so very generally adopted that we are apt to forget its inventor and the credit that belongs to our school.

In Vol. XLIV, No. 164 (1927), we find the address delivered by Mr. F. Richardson Cross on the occasion of the fiftieth anniversary of the foundation of University College, entitled "Early Medical Teaching in Bristol."

The almost sudden death on 9th December, 1932, of the Assistant Editor, Dr. Carey Coombs, at the age of 54, was so great a loss to the Bristol Medical School and, indeed, to the profession the world over, as to appear a real tragedy. For what he was and what he had accomplished we may refer to the obituary notice in the *Journal*. It has been our privilege to publish many of his valuable contributions to cardiology, among which, perhaps, stand out his Long Fox Lecture (1926), "The Ætiology of Cardiac

Disease," and particularly "Thirty Years' Progress in the Study of Rheumatic Heart Disease," published posthumously in our last issue. What we could say here would inevitably appear inadequate to convey the high esteem in which Carey Coombs was held. Looking back through over fifty years one finds but few of our colleagues who have been accorded an individual memorial; but to perpetuate the work, and especially to furnish opportunity for others to carry on the work, that Carey Coombs initiated is a duty; and we cannot doubt that the efforts of the organizing Committee will be crowned with complete success.

The office of Assistant Editor was filled by the appointment of Mr. E. Watson-Williams. The *Journal* has undergone no further outward changes of moment, but has continued to grow in strength and usefulness, and in the support of members and subscribers. The *Journal* of to-day requires no words of commendation, simply because we all know it. But as this history will be a record to which in time to come our colleagues of quite another generation may turn, they should know that the present Editor and his collaborators, who have established and enhanced the *Journal's* reputation and standing, all bear the honourable scars of the Great War that will then be but history.

### THE MEDICAL LIBRARY

In connection with the development of the Medical Library, the Bristol Medical School owes to the Society and its *Journal* a debt that it can never hope to repay. The actual foundation of the Library, as we know it, and its successful progress, are due almost entirely to the energy and enthusiasm of Mr. J. Greig Smith and Mr. L. M. Griffiths.

In his Presidential Address[1] the former tells us: "I do more than care for the success of the Library; my heart and soul are in it. I am, indeed, committed to it; for when the *Journal* began to earn money, and there was a likelihood of our generous Committee distributing it too liberally to charities, I pleaded for the hoarding of it—that we might buy books and found a library. These small hoardings accumulated and they made possible the existence of a Medical Library in our midst. The valuable and extensive Infirmary Library will be placed on our shelves; the Hospital will lend its books. With some 10,000 volumes on our shelves and some 90 current periodicals on the tables, we may fairly claim to have made a good start."

One of the most fruitful means of keeping the Library up to date by the addition of newly-published works has been the Review service of the *Journal* from the very beginning. Already by July, 1883, six newly-published works had been received for review, and six more within the first year; within the three years 1884, 1885, and 1886 no less than 102 books were received and reviewed.

A moment's reflection tells us that the sending of these works must have been in response to the Editor's efforts to start at once in establishing Reviews as an important feature of the *Journal's* work. But without L. M. Griffiths, who was a born librarian, to support him, to care for and arrange the books, the success of this feature would have been far less, and it was certainly due to the organizing genius of Griffiths that as years went by the Library was in receipt of a wonderfully valuable exchange list which, in 1913, amounted to 112 current medical periodicals.

[1] *Journal*, December, 1893, vol. xi., p. 216.

In 1921 the combined Medical Libraries contained 24,659 volumes, 9,278 belonging to the University and 15,381 to the Society. In 1926 the *Journal* records the gift of the Society's valuable Medical Library to the University and the allocation of a room for the use of members of the Society in the Library wing of the new University Building. To-day it contains 24,000 volumes (excluding duplicates, obsolete editions and unbound books), increasing 400–500 annually; it is particularly rich in complete series of medical periodicals, and has been authoritatively ranked as one of the best Medical Libraries in the provinces. The *Journal* continues to play no insignificant part in this development. Of the new books (excluding periodicals) each year added to the Library rather more than half come through the Review service of the *Journal*, and of the 175 periodicals now being received 70 come in exchange for the *Journal*.

### FORMAT

Vol. I, 1883—Two issues. Demy octavo; page 22 ems wide, 33 lines $=6\frac{7}{8}$ in. deep; small pica old style; Reviews, etc., long primer modern; illustrations from drawings on stone and photographs pasted in. Cover pale green.

Vol. II, 1884 and subsequently, quarterly issue.

1892—Half-tone blocks for illustrations.

1893—Page 23 ems wide, 38 lines $=6\frac{7}{8}$ in. deep; long primer old style.

1907—Type first set by monotype in 10 point old style.

1914—Type enlarged to 11 point old style; Reviews in 10 point old style.

1915—Three numbers only. 1916—Two numbers. 1917—Three numbers.

Vol. XXXVI—One number for 1918 and two for 1919. Vol. XXXVII, 1920—Four numbers. 1921—Three numbers. 1922—Two numbers. 1923—Four numbers.

1925—No. 156. Special number to commemorate Royal opening of new University buildings; on art paper. Cover changed to dark neutral tint.

1926—Vol. XLIII, No. 159. Royal octavo; page 24 ems wide, 35 lines=$7\frac{1}{8}$ in. deep; 12 point modern; Reviews, etc., 11 point modern.

### EDITORIAL STAFF.

From 1883 to the formation of the Committee in 1891 the publication of the *Journal* was entirely the work of J. Greig Smith and L. M. Griffiths.

*Editor.*

| | |
|---|---|
| J. Greig Smith .. | 1883–1892 |
| R. Shingleton Smith | 1892–1912 |
| P. Watson-Williams | 1912–1926 |
| [1]J. A. Nixon .. .. | 1926 |

*Editorial Secretary.*

| | |
|---|---|
| B. M. H. Rogers .. | 1895–1898 |
| J. Taylor .. .. | 1899–1909 |
| J. A. Nixon .. .. | 1909–1912 |
| J. M. Fortescue-Brickdale | 1913–1921 |
| [3]W. A. Smith .. .. | 1915–1918 |
| [1]A. L. Flemming .. | 1921 |

*Assistant Editor.*

| | |
|---|---|
| [2]L. M. Griffiths .. | 1883–1899 |
| P. Watson-Williams | 1900–1912 |
| J. A. Nixon .. .. | 1912–1926 |
| Carey F. Coombs .. | 1926–1932 |
| [1]E. Watson-Williams | 1933 |

*Treasurer of the Medico-Chirurgical Society, ex-officio.*

| | |
|---|---|
| [1]A. E. Iles .. .. | 1926 |

*Committee.*

| | |
|---|---|
| Barclay J. Baron .. | 1891–1893 |
| J. Michell Clarke | 1891–1918 |
| F. R. Cross .. .. | 1891–1898 |
| W. H. Harsant .. | 1891–1899 |
| J. Swain .. .. .. | 1897–1924 |
| B. M. H. Rogers .. | 1898–1900 |
| J. Lacy Firth .. | 1900–1926 |
| Carey F. Coombs .. | 1919–1926 |
| [1]E. W. Hey Groves | 1924 |
| E. Watson-Williams | 1926–1933 |
| [1]A. Rendle Short .. | 1933 |

[1] Present Committee. [2] And Publications Secretary, 1883–1895.
[3] During absence of Captain Fortescue-Brickdale on service.

*The Arrowsmith Colophon.*

## Figures and Tables

**Figure f1:**
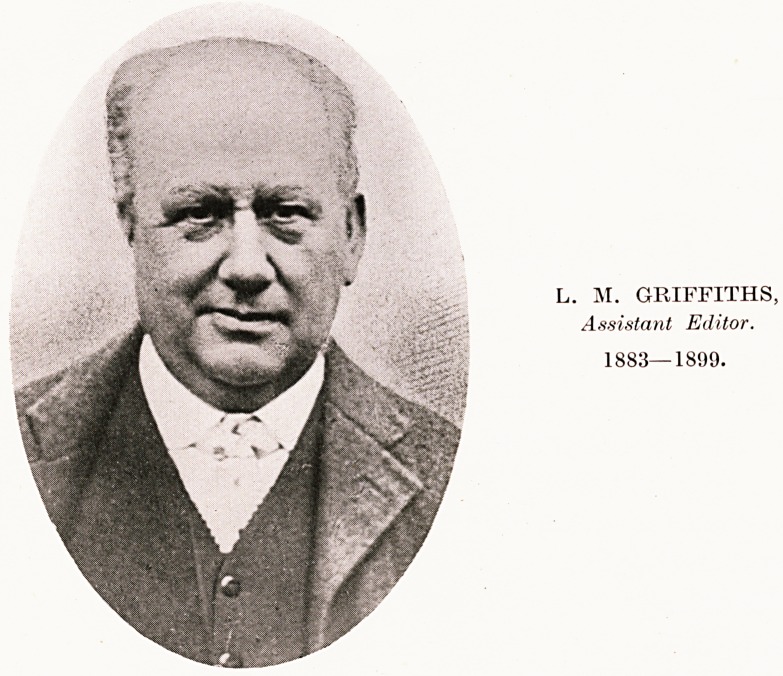


**Figure f2:**